# Endodontic Treatment of Bilateral Maxillary First Premolars with Three Roots Using CBCT: A Case Report

**DOI:** 10.1155/2014/505676

**Published:** 2014-03-04

**Authors:** Sujatha Gopal, Gijo John, K. Pavan Kumar, Swarna Latha, Suma Latha, Sowmya Kallepalli

**Affiliations:** Department of Conservative Dentistry and Endodontics, MNR Dental College & Hospital, Sangareddy, Andhra Pradesh 502 294, India

## Abstract

One of the determining factors for the success of endodontic therapy is understanding the morphological anatomy of the tooth structure and its variants in relation to its template anatomy. The internal anatomy of maxillary first premolars is particularly complex due to their variation in number of roots and canal configuration. However, the bilateral presence of three roots in a maxillary first premolar is of rare occurrence. This case report describes the unusual anatomy bilaterally detected in maxillary first premolars using Cone-Beam Computed Tomography.

## 1. Introduction

Thorough knowledge of the internal anatomy of teeth is essential before an operator can rationally approach any endodontic procedure. The anatomical and structural complexities of the root canal should be effectively assessed and efficiently approached for successful endodontic therapy. An extra root is an additional challenge which begins at case assessment and involves all operative stages, including cavity design, canal access, localization, cleaning and shaping of the root canal system, and proper obturation [1]. The internal anatomy of the maxillary first premolar is particularly complex due to its variation in number of roots and canal configuration [2]. In the case of maxillary first premolar, three root canals are found at a frequency of 0.5–6% [3, 4].

## 2. Case Report

A 34-year-old female patient has reported to the Department of Conservative Dentistry and Endodontics, MNR Dental College and Hospital, Sangareddy, with a chief complaint of pain in the upper right and left back tooth region for a week. Her medical history was noncontributory. Clinical examination of the area of chief complaint revealed dental caries with tenderness on percussion.

**Figure 1:**
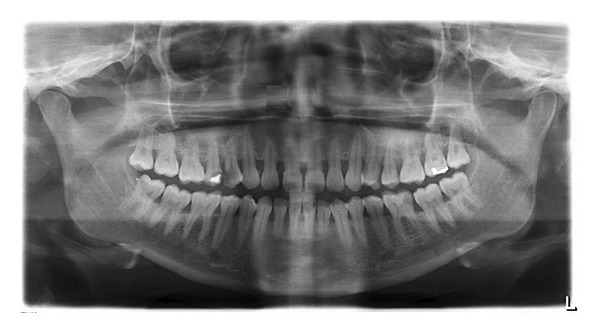
OPG showing three rooted bilateral first premolar; mesiodistal root diameter was greater than the mesiodistal width of the crown.

**Figure 2:**
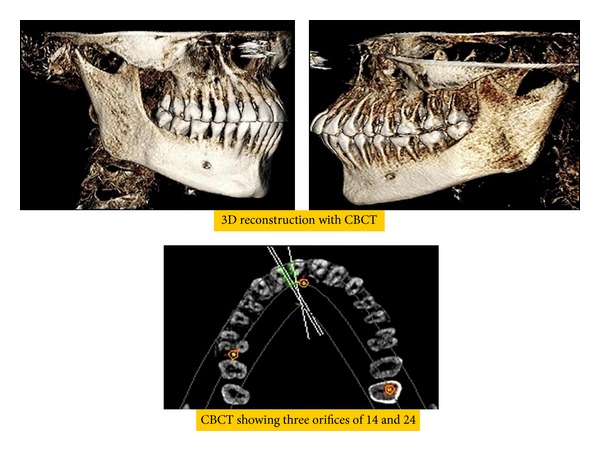
CBCT images of 14 and 24.

Based on clinical findings, radiographic interpretation, and vitality tests, a diagnosis of acute apical periodontitis in relation to right and left maxillary first premolar was made and endodontic therapy was planned. On further examination of the radiographs, an abrupt loss of radiolucency in the pulp canal was noticed in relation to right and left first premolar region. The mesiodistal root diameter was greater than the mesiodistal width of the crown (Figure 1). With these findings, a possible anatomical tooth variation was suspected in relation to right and left first premolar region and reconfirmed using Cone-Beam Computed Tomography (Gentex), Figure 2.

The right maxillary first premolar was anaesthetized using 2% lignocaine with 1 : 80,000 adrenaline (Lignox) and isolated with rubber dam. The endodontic access opening was prepared. The access cavity was modified with a cut at the buccoproximal angle from the entrance of the buccal canals to the cavosurface angle resulting in a cavity with a T-shaped outline [5]. The mesiobuccal, distobuccal, and the palatal canals were explored with a size 10 K—file (Mani). A working length was established with an apex locator (I-Pex).

**Figure 3:**
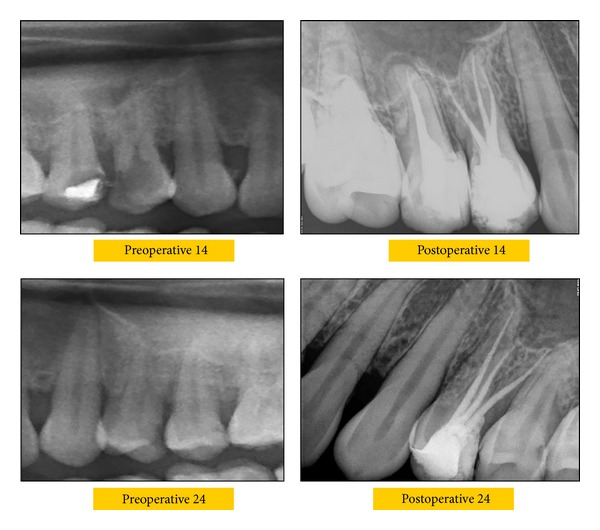
Preoperative and postoperative radiographs of 14 and 24 showing three roots.

A working length radiograph confirmed a type VIII Vertucci root canal morphology [6]. Cleaning and shaping were performed using crown down technique with ProTaper rotary instruments (Dentsply) under abundant irrigation with 3% sodium hypochlorite solution. All canals were enlarged to size F2. Final irrigation was done with 17% EDTA and the root canal space was obturated with gutta percha and resin sealer (AH Plus, Dentsply) by lateral condensation. The coronal access was restored with resin composite (3M ESPE). Similarly, left maxillary first premolar tooth was diagnosed with having three independent roots and root canals and endodontic therapy was performed in the same manner as explained above (Figure 3).

## 3. Discussion

The anatomical abnormalities of the root canal systems are a challenge to an operator for a successful endodontic therapy. A major reason for a failed root canal treatment is undetected extra roots and canals [7]. Three rooted maxillary premolars were reported to be a rare variation in Asian population (0.6%) as compared to non-Asian population [8].

Three rooted premolars were mostly identified bilaterally [1]. In the case report discussed, the left and right maxillary first premolars have type VIII root canal morphology according to Vertucci classification [6].

The anatomy of maxillary premolars with three root canals, mesiobuccal, distobuccal, and palatal, is similar to that of adjacent maxillary molars and they are therefore sometimes called “small molars” or “ridiculous” [9]. When a preoperative radiograph revealed an atypical tooth shape and unusual contour, further radiographs should be taken with different angulations to confirm any unusual anatomical features [4]. When there is an abrupt straightening or loss of a radiolucent canal in the pulp cavity, an extra canal should be suspected in the same root or in the other independent roots [4, 10]. The presence of an eccentric orifice other than in its normal location in premolars buccopalatally leads to the suspicion of the presence of an extra canal [11].

Whenever the mesiodistal width of the midroot region is equal to or greater than the mesiodistal width of the crowns and palpation of buccal cervical area of the tooth with an explorer reveals a buccal furcation, the tooth is likely to have extra roots [3, 10]. Diagnostic measures such as multiple preoperative radiographs, examination of the pulp chamber floor with a sharp endodontic explorer, toughing of grooves with ultrasonic tips, staining the chamber floor with 1% methylene blue dye, performing the sodium hypochlorite champagne test and visualizing canal bleeding points transillumination, white line test, red line test, and advanced radiographic methods are important aid in locating root canal orifices.

With newer technology, various diagnostic aids have been introduced which assist in identifying the variations and root canal anatomy.

In this case, Cone-Beam Computed Tomography was used which is an advancement in CT imaging. CBCT is capable of providing images of high diagnostic quality with shorter scanning times and lower dosages compared to those of conventional CT Scans [12]. CBCT is a practical tool for noninvasive and three-dimensional reconstruction imaging for use in morphologic analysis and endodontic application [13].

## 4. Conclusion

Thorough knowledge of the root canal anatomy, careful interpretation of the angled radiographs, proper endodontic access cavity preparation, and exploration of the root canal are the prerequisites for endodontic success. The maxillary first premolars, without a doubt, are among the most difficult teeth to be treated endodontically for various reasons: the number of roots, the number of canals, the direction and longitudinal depressions of the roots, the various pulp cavity configurations, and also the difficulties in visualizing the apical limit by radiographs.
